# Impact of different EMA/CO treatment regimens in women with gestational trophoblastic neoplasia: brazilian multicenter retrospective cohort study

**DOI:** 10.61622/rbgo/2026rbgo52

**Published:** 2026-07-17

**Authors:** Laís Cristhine Santos Souza, Michelle Samora de Almeida, Antonio Braga, José Mauro Madi, Edward Araujo Júnior, Alberto Borges Peixoto, Marcio Bezerra Barcellos, Lucas Ribeiro Borges de Carvalho, Roney Cesar Signorini, Sue Yazaki Sun

**Affiliations:** 1 Universidade Federal de São Paulo Escola Paulista de Medicina São Paulo SP Brazil Escola Paulista de Medicina, Universidade Federal de São Paulo, São Paulo, SP, Brazil; 2 Universidade Federal do Rio de Janeiro Rio de Janeiro RJ Brazil Universidade Federal do Rio de Janeiro, Rio de Janeiro, RJ, Brazil; 3 Universidade de Caxias do Sul Caxias do Sul RS Brazil Universidade de Caxias do Sul, Caxias do Sul, RS, Brazil; 4 Universidade Federal do Triângulo Mineiro Uberaba MG Brazil Universidade Federal do Triângulo Mineiro, Uberaba, MG, Brazil; 5 Faculdade de Medicina de Petrópolis Petrópolis RJ Brazil Faculdade de Medicina de Petrópolis, Petrópolis, RJ, Brazil

**Keywords:** Gestational trophoblastic disease, Etoposide, Methotrexate, Dactinomycin, Vincristine, Drug resistance, neoplasm, Toxicity

## Abstract

**Objective:**

To compare the clinical outcome of women with gestational trophoblastic neoplasia (GTN) treated with the conventional EMA/CO (etoposide, methotrexate, actinomycin-D, cyclophosphamide, vincristine) with those treated with the modified EM/CO regimen.

**Methods:**

Brazilian multicenter retrospective cohort study evaluated medical records of women diagnosed with GTN who were treated with the standard EMA/CO or with the modified regimen in which actinomycin-D was suppressed (EM/CO). The primary outcome was the occurrence of remission following chemotherapy treatment.

**Results:**

Remission rate with EMA/CO was 85.3%, with 14.7% of women showing chemoresistance, while with EM/CO, the remission rate was 72.7%, with 27.3% of women chemoresistance, without statistical difference between the groups. A total of 142 women were analyzed, of whom 109 were treated with EMA/CO and 33 received EM/CO. Women who used the EM/CO had higher prevalence of invasive mole (51.5% vs. 14.7%, p<0.0001), lower prevalence of choriocarcinoma (9.1% vs. 26.6%, p=0.035), and lower prevalence of metastases (33.3% vs. 61.5%, p=0.004) compared to women who received the EMA/CO. Women with risk score ≥ 7 had higher prevalence of recurrence (61.5% vs 10.0%, p=0.005) and chemoresistance using the EM/CO (53.8% vs 10.0%, p=0.013) compared to women with score ≤ 6. The pre-treatment serum hCG levels was a moderately significant predictor (AUC: 0.77, 95%CI 0.66-0.88, p<0.0001) for identifying chemoresistance. The number of EM/CO cycles was a strong significant predictor (AUC: 0.81, 95%CI 0.66-0.96, p<0.0001) for identifying toxicity to chemotherapy.

**Conclusion:**

EM/CO had higher prevalence of need for second-line treatment, and chemoresistance.

## Introduction

Gestational trophoblastic neoplasia (GTN) refers to malignant lesions derived from chorionic villi and extravillous trophoblast, including invasive mole, choriocarcinoma, placental site trophoblastic tumor, and epithelioid trophoblastic tumor.^(1-4)^ Approximately 75% of gestational trophoblastic neoplasia cases arise after a molar pregnancy, while the remaining cases occur following spontaneous abortion, ectopic pregnancy, or term/preterm pregnancy.^(1,3)^ The rarer placental site trophoblastic tumor and epithelioid trophoblastic tumor are associated with term pregnancies or non-molar miscarriages in 95% of cases.^(5)^

Invasive mole and choriocarcinoma represent the majority of GTN cases. These tumors are characterized by high levels of human chorionic gonadotropin (hCG) and excellent sensitivity to chemotherapy, with cure rates exceeding 90%.^(3)^ In contrast, placental site trophoblastic tumor and epithelioid trophoblastic tumor produce lower hCG levels and are relatively resistant to chemotherapy, making surgery the preferred treatment in non-metastatic cases.^(3,6)^

Clinical presentation varies with the antecedent pregnancy and disease extent.^(1,3)^ While an enlarged uterus, irregular bleeding, and thecalutein cysts may suggest GTN ^(3)^, more than 50% of post-molar GTN cases are diagnosed solely by hCG plateau or rise following uterine evacuation.^(7)^ Treatment is determined by the FIGO risk score, which predicts resistance to single-agent chemotherapy ^(3,4)^, with cure rates above 90% even in disseminated disease.^(1,3,8)^

Low-risk GTN (FIGO score ≤6) is managed with methotrexate or actinomycin-D ^(1,4,9,10)^, showing remission rates between 50% and 90%.^(4)^ Hysterectomy may be considered in selected cases alongside adjuvant chemotherapy to eliminate occult metastases.(9) High-risk GTN (FIGO score ≥7 or stage IV) requires multi-agent chemotherapy and possibly surgery or radiotherapy.^(1-3)^ The EMA/CO regimen (etoposide, methotrexate, actinomycin-D, cyclophosphamide, vincristine) is widely used in this context due to its favorable toxicity profile and high remission and survival rates, with reported primary remission rates between 54% and 91%.^(1-4)^

However, since 2013, Brazil has faced intermittent shortages of actinomycin-D, critically affecting the treatment of methotrexate-resistant GTN and cases requiring EMA/CO. As noted by Melo and Paulino ^(11)^, the withdrawal of this essential drug has disrupted both public and private healthcare services, leading to treatment delays and increased use of less effective alternatives. Despite efforts by national medical associations, the persistent unavailability of actinomycin-D has led to the adoption of alternative regimens. In low-risk GTN, protocols with carboplatin and etoposide have been used. In patients requiring EMA/CO, actinomycin-D has been omitted, and patients have instead received the modified EM/CO regimen. Winter et al.^(12)^ demonstrated that single-agent carboplatin achieved an 81% remission rate in methotrexate-resistant low-risk GTN, with myelosuppression as the main toxicity. However, no studies have evaluated the clinical outcomes of omitting actinomycin-D from the EMA/CO regimen.

The aim of this study was to compare the clinical outcomes of women with low- and high-risk GTN treated with the conventional EMA/CO regimen versus the modified EM/CO regimen.

## Methods

A Brazilian multicenter retrospective cohort study evaluated medical records of women diagnosed with GTN who were treated with the standard EMA/CO regimen or with the modified regimen in which actinomycin-D was suppressed (EM/CO) between January 2010 and December 2020. These women were from three Brazilian Reference Centers of Gestational Trophoblastic Diseases (Universidade Federal de São Paulo, Universidade Federal do Rio de Janeiro and Universidade de Caxias do Sul).

Women diagnosed with GTN according to the FIGO 2000 criteria,^(10)^ treated with the standard (EMA/CO) or modified (EM/CO) regimen, and followed for at least 12 months were included. Women with a diagnosis of GTN whose pathology was placental site trophoblastic tumor or epithelioid trophoblastic tumor, or who became pregnant during follow-up or discontinued follow-up before 12 months were excluded.

All patients included in the study received multiagent chemotherapy as the first-line treatment. For low-risk patients, this was initiated after failure of single-agent therapy. High-risk patients received multiagent chemotherapy as the initial treatment for GTN. Low-risk GTN was defined as patients with a WHO/FIGO prognostic score of ≤6, while high-risk GTN was defined as patients with a score of ≥7, following the criteria established by the WHO scoring system. This classification considers factors such as age, antecedent pregnancy, interval from antecedent pregnancy, pre-treatment serum hCG levels, largest tumor size, sites of metastases, number of metastases, and prior failed chemotherapy regimens.

The standard EMA/CO regimen included the administration of etoposide (100 mg/m^2^) and methotrexate (300 mg/m^2^) intravenously on day 1, followed by actinomycin-D (0.5 mg) intravenously on days 1 and 2. Cyclophosphamide (600 mg/m^2^) and vincristine (1 mg/m^2^) were administered intravenously on day 8. This cycle was repeated every 14 days. In the modified EM/CO regimen, actinomycin-D was excluded, and the remaining drugs were administered according to the same schedule and dosages as EMA/CO (Supplementary material). Details are summarized in table 1s.

Quantitative serum hCG levels are the diagnostic mainstay of GTN, the diagnostic criteria for which are presented as follow: 1) Four or more hCG plateau levels over a period of more than 3 weeks, i.e. on days 1, 7, 14 and 21; 2) Elevated hCG levels on 3 or more consecutive measurements over a period of at least 2 weeks, i.e. on days 1, 7 and 14; 3) Histologic diagnosis of choriocarcinoma; 4) Elevated hCG levels for 6 months or more.^(10)^

A plateau is defined as less than a 10% change in hCG values over three consecutive cycles, while an increase is defined as a change in hCG levels of more than 10% over two consecutive cycles.^(15)^ In this study, chemotherapy resistance is defined as a plateau or increase in serum hCG levels and/or the development of new metastases.^(13,14)^ After remission, monthly follow-up was conducted for 12 consecutive months, as recommended in the literature.^(16)^

Recurrence was diagnosed as a re-elevation of serum hCG levels after achieving remission (three normal hCG values), with no evidence of a new pregnancy and after excluding other possible causes of hCG elevation, such as pituitary hCG production, false-positive results, or other malignancies.^(15)^

The primary outcome was the occurrence of remission following chemotherapy treatment with the two regimens studied, EMA/CO and EM/CO. The secondary outcomes were the occurrence of adverse events, median number of cycles, resistance, recurrence and death in women treated with the two regimens studied, EMA/CO and EM/CO.

The following variables were analyzed: age, number of pregnancies, number of deliveries and number of miscarriages. With regard to the clinical variables, pre-treatment serum hCG levels, anatomopathologic result, origin of GTN (hydatidiform mole, full-term/preterm pregnancy, miscarriage, ectopic pregnancy), the staging of the neoplasm (I - neoplasm confined to the uterus, II - neoplasm with pelvic metastases, III - neoplasm with lung metastases, IV - other organs involved), FIGO prognostic risk factor, interval between the pregnancy that led to GTN and the start of chemotherapy, presence of metastases and their locations, regimen used (EMA/CO or EM/CO).

Regarding the therapeutic variables for GTN, it was evaluated the occurrence of remission after treatment, defined as the normalization of hCG levels < 5,000 mU/l and maintained for at least 4 weeks; indication for chemotherapy (treatment of chemoresistant low- or high-risk GTN); number of cycles required to achieve remission; toxicity according to the Common Terminology Criteria for Adverse Events version 5.0 2017 (CTCAE, 2017)^(17)^; total number of chemotherapy cycles for remission; occurrence of recurrence, defined as re-elevation of serum hCG levels after 3 normal measurements, in the absence of new pregnancy; occurrence of death related to the chemotherapy used, disease-free survival and overall survival.

The data were collected in an Excel 2007 spreadsheet (Microsoft Corp., Redmond, WA, USA) and analyzed using SPSS version 20.0 (SPSS Inc., Chicago, IL, USA) and Prisma GraphPad (San Diego, CA, USA). The Shapiro-Wilk normality test was used to analyze whether the values had a Gaussian distribution. Non-parametrically distributed variables were presented as medians and minimum and maximum values. Parametric variables were presented as mean and standard deviation. Categorical variables were described as absolute and percentage frequencies. The Mann-Whitney test was used to compare non-parametrically distributed continuous variables between groups. Student's t-test was used to compare continuous variables with parametric distribution between groups. Chi-squared test was used to assess the association between groups and categorical variables. Binary logistic regression was used to calculate the odds ratio (OR) for outcomes such as chemoresistance and toxicity. Binary stepwise forward logistic regression was then used to evaluate the predictive model for chemoresistance and toxicity. A receiver operating characteristics (ROC) curve was used to determine the best cut-off of pre-treatment serum hCG level and number of chemotherapy cycles in women with GTN to predict chemoresistance and toxicity to treatment.

This study was approval by the Ethics Committee of Universidade Federal de São Paulo (Process nº 5.875.065 of February 03, 2023). Written informed consent was waived due to the anonymous retrospective nature of the study. All research procedures complied with the 1964 Helsinki Declaration and its later amendments or ethical standards.

## Results

**Figure 1:**
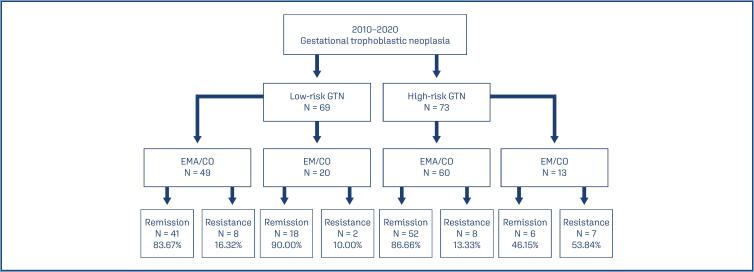
Flowchart of the studied population

We observed a remission rate with EMA/CO of approximately 85.3%, with 14.7% of women showing chemoresistance, while in the group of women who received EM/CO, the remission rate was 72.7%, with 27.3% of women showing chemoresistance, with no statistical significance between the groups. A total of 142 women were analyzed, of whom 109 were treated with EMA/CO and 33 received EM/CO. Of the women treated with EMA/CO, 60 were high- and 49 were low-risk GTN, while in the group of women receiving EM/CO, 13 were high- and 20 were low-risk GTN (Figure 1).

Women who used the EM/CO chemotherapy regimen had a higher prevalence of invasive mola (51.5% vs. 14.7%, p<0.0001), lower prevalence of choriocarcinoma (9.1% vs. 26.6%, p=0.035), lower prevalence of previous full-term pregnancy (3.0% vs. 19.3%, p=0.026), and lower prevalence of metastases (33.3% vs. 61.5%, p=0.004) compared to women who received the EMA/CO chemotherapy regimen (Table 1).

**Table 1:** Clinical characteristics of the studied population

		EM/CO (N=33)	EMA/CO (N=109)	p-value
Age (years)		30.0 (21.0-48.0)	32.0 (14.0-54.0)	0.092 *
Number of pregnancies		3.0 (1.0-7.0)	2.0 (1.0-8.0)	0.338*
Number of deliveries		1.0 (0.0-5.0)	1.0 (0.0-7.0)	0.279*
Miscarriage		1.0 (0.0-2.0)	0.0 (0.0-5.0)	0.408 *
Serum hCG pre-treatment levels (mU/ml)		71000.0 (41.0-900000.0)	110000.0 (1123.0-1838069.0)	0.084 *
Pathology in diagnosis				
	No histopathology	39.4% (13/33)	58.7 % (64/109)	0.072 **
	Invasive mole	51.5% (17/33)	14.7% (16/109)	< 0.0001 **
	Choriocarcinoma	9.1% (3/33)	26.6% (29/109)	0.035 **
Previous pregnancy				
	Molar pregnancy	72.7% (24/33)	71.6% (78/109)	> 0.999 **
	Miscarriage	21.2% (7/33)	8.3% (9/109)	0.056 **
	Ectopic pregnancy	3.0% (1/33)	0.9% (1/109)	0.421 **
	Full-term pregnancy	3.0% (1/33)	19.3% (21/109)	0.026 **
Clinical stage				
	I	63.6% (21/33)	39.4% (43/109)	0.017 **
	II	3.0% (9/33)	15.6% (17/109)	0.196 **
	III	27.3% (9/33)	36.7% (40/109)	0.404 **
	IV	6.1% (2/33)	8.3% (9/109)	> 0.999 **
Prognosis score				
	≤ 6	60.6% (20/33)	45.0% (49/109)	0.163 **
	≥ 7	39.4% (13/33)	55.0% (60/109)	0.163^**^
Pregnancy and chemotherapy (weeks)		10.0 (3.0-192.0)	20.0 (4.0-104.0)	0.323 *
Metastases		33.3% (11/33)	61.5% (67/109)	0.004 **
Site of metastases				
	Lungs	21.2% (7/33)	38.5% (42/109)	0.093 **
	Vagina	0.0% (0/33)	10.1% (11/109)	0.067 **
	Brain	0.0% (0/33)	1.8% (2/109)	> 0.999 **
	Liver	0.0% (0/33)	2.8% (3/109)	> 0.999 **
	Other	3.0% (1/33)	5.5% (6/109)	> 0.999 **
	Lungs and brain	9.1% (3/33)	2.8% (3/109)	0.138 **
	No metastases	66.7% (22/33)	38.5% (42/109)	0.005 **

Mann-Whitney

* median (minimum-maximum); Chi-squared

** % (n/N). p<0.05

Women who received first line EM/CO had lower prevalence of not using any second-line regimen (69.7% vs. 88.1%, p=0.027), higher prevalence of using the EP/EM regimen (18.2% vs. 0.0%, p=0001) and higher prevalence of using the EP/EM + TE/TP regimen (9.1% vs. 0.0%, p=0.011) (Table 2). There was no association between chemotherapy regimens and toxicity (p=0.124) and side effects (Table 2S).

**Table 2:** Association between chemotherapy regimen used in the treatment of women with gestational trophoblastic neoplasia and clinical outcome

		EM/CO (N=33)	EMA/CO (N=109)	p-value
Number of cycles (first-line treatment)		5.0 (1.0-12.0)	7.0 (1.0-14.0)	0.291 *
Resistance to chemotherapy		27.3% (9/33)	14.7% (16/109)	0.096**
Second-line treatment				
No chemotherapy		69.7% (23/33)	88.1% (96/109)	0.027 **
EP/EM		18.2% (6/33)	0.0% (0/109)	0.0001 **
EP/EM + TE/TP		9.1% (3/33)	0.0% (0/109)	0.011 **
EP/EMA		0.0% (0/33)	4.6% (5/109)	0.509 **
EP/EMA + TE/TP		0.0% (0/33)	6.4% (7/109)	0.200 **
TE/TP + ICE		3.0 % (1/33)	0.0% (0/109)	0.232 **
TE/TP + Pembrolizumab		0.0% (0/33)	0.9% (1/109)	>0.999 **
Recurrence		12.1% (4/33)	6.4% (7/109)	0.283 **
Death		6.1% (2/33)	5.5% (6/109)	0.903 **
Age at death (years)		31 (29-33)	34 (15-54)	0.285 *
Interval between starting chemotherapy and death (weeks)		19.5 (1-38)	5.0 (2-108)	0.607 *
Cause of death				
Vaginal bleeding		0.0% (0/33)	1.8% (2/109)	>0.999 **
Respiratory failure		3.0% (1/33)	3.7% (4/109)	>0.999 **
Brain metastases		3.0 % (1/33)	0.0% (0/109)	0.232 **
	N/A	93.9% (31/33)	94.5% (103/109)	>0.999 **

EP/EM: etoposide, platinum, etoposide and methotrexate; TE/TP: paclitaxel, etoposide, paclitaxel and platinum; EP/EMA: etoposide, platinum, etoposide, methotrexate and actinomycin-d; ICE: ifosfamide, cyclophosphamide and etoposide; N/A: not available. Mann-Whitney

* median (minimum-maximum); Chi-squared

** % (n/N). p<0.05

Women with a score ≥ 7 had higher mean number of chemotherapy cycles than women with score ≤ 6 (7.4 vs 4.8 cycles, p=0.021). It was observed that women with risk score ≥ 7 had higher prevalence of recurrence (61.5% vs 10.0%, p=0.005) and chemoresistance (53.8% vs 10.0%, p=0.013) using the EM/CO regimen compared to women with score ≤ 6. Women with score ≥ 7 using the EM/CO regimen were 14.4 (OR: 14.4, 95%CI 2.46-75.68, p=0.005) and 10.5 (OR: 10.5, 95%CI 1.79-55.6, p=0.013) more likely to require second-line treatment and develop chemoresistance, respectively, than women with score ≤ 6 (Table 3).

**Table 3:** Influence of prognostic score and chemotherapy regimen used.

		Score ≥7 (N=73)	Score ≤ 6 (N=69)	OR (95%CI)	p-value
Number of cycles (first-line treatment)					
	EM/CO	7.4 (3.5)	4.8 (2.6)		0.021 *
	EMA/CO	6.8 (3.1)	6.0 (2.4)		0.159 *
Second-line treatment					
	EM/CO	61.5% (8/13)	10.0% (2/20)	14.4 (2.46-75.68)	0.005 **
	EMA/CO	8.3% (5/60)	16.3% (8/49)	0.46 (0.16-1.42)	0.242 **
Chemoresistance					
	EM/CO	53.8% (7/13)	10.0% (2/20)	10.5 (1.79-55.61)	0.013 **
	EMA/CO	13.3% (8/60)	16.3% (8/49)	0.8 (0.26-2.38)	0.787 **
Recurrence					
	EM/CO	15.4% (2/13)	10.0% (2/20)	1.6 (0.22-11.4)	>0.999 **
	EMA/CO	5.0% (3/60)	8.2% (4/49)	0.6 (0.14-2.30)	0.698 **
Toxicity					
	EM/CO	92.3% (12/13)	80.0% (16/20)	3.0 (0.37-39.4)	0.625 **
	EMA/CO	73.3% (44/60)	69.4 (34/49)	1.2 (0.52-2.77)	0.674 **
Death					
	EM/CO	15.4% (2/13)	0.0% (0/20)	Infinite (0.73-infinite)	0.147 **
	EMA/CO	8.3% (5/60)	2.0% (1/49)	4.36 (0.55-52.3)	0.220 **

OR: odds ratio; CI: confidence interval. Student's t-test

* mean (standard deviation); Chi-squared

** % (n/N); binary logistic regression. p<0.05

Forward stepwise binary logistic regression analysis was conducted to identify the most significant predictors of chemoresistance to EMA/CO and EM/CO regimens. The covariates included in the model were a score ≥ 7, invasive mole, clinical stage I, choriocarcinoma, and the presence of metastases. For women treated with EM/CO, the presence of metastases emerged as a significant predictor, increasing the risk of chemoresistance by 9.0 times (OR: 9.0, 95% CI 1.02-79.61, p=0.047). Interestingly, a score ≥ 7 lost statistical significance when metastases were introduced into the model (OR: 3.41, 95%CI 0.37-31.0-55.6, p=0.276). In contrast, for women treated with EMA/CO, none of the covariates, including score ≥ 7 (p=0.787), invasive mole (p=0.620), choriocarcinoma (p=0.100), clinical stage I (p=0.863), and presence of metastases (p=0.519), were found to be significant predictors of chemoresistance. Considering only women with a score ≥ 7, the influence of the type of first-line chemotherapy regimen on the number of cycles, need for second-line treatment, chemoresistance, toxicity, recurrence and death was evaluated. It was observed that women who used EM/CO had a higher prevalence of need for second-line treatment (61.5% vs 8.3%, p<0.0001) and chemoresistance (53.8% vs 13.3%, p=0.0034) when compared to women who used EMA/CO. Women using the EM/CO regimen were 17.6 (OR: 17.6, 95%CI 3.95-64.27, p<0.0001) and 7.6 (OR: 7.6, 95%CI 1.92-25.05, p=0.0034) more likely to require second-line treatment and develop chemoresistance, respectively, than women using EMA/CO (Table 4).

**Table 4:** Influence of the chemotherapy regimen used during the first-line treatment in women with a score ≥ 7

	EM/CO (N=13)	EMA/CO (N=60)	OR (95%CI)	p-value
Number of cycles (first-line treatment)	7.4 (3.5)	6.8 (3.1)		0.536 *
Second line treatment	61.5% (8/13)	8.3 % (5/60)	17.6 (3.95-64.27)	<0.0001 **
Chemoresistance	53.8% (7/13)	13.3% (8/60)	7.6 (1.92-25.05)	0.0034 **
Recurrence	15.4% (2/13)	5.0% (3/60)	3.4 (0.55-18.14)	0.214 **
Toxicity	92.3% (12/13)	73.3% (44/60)	4.4 (0.63-49.42)	0.275 **
Death	15.4% (2/13)	8.3% (5/60)	2.0 (0.35-11.99)	0.600 **

OR: odds ratio; CI: confidence interval. Student's t-test

* mean (standard deviation); Chi-squared

** % (n/N); binary logistic regression. p<0.05

Considering only the women with a score ≤ 6, it was evaluated the influence of the type of chemotherapy regimen used in the first-line treatment on the number of cycles, need for second-line treatment, chemoresistance, toxicity, recurrence, and death. There was no significant effect of the first-line chemotherapy regimen on any of the variables analyzed (Table 3S).

A forward stepwise binary logistic regression model was employed to identify the best predictors of chemoresistance in women with a score ≤ 6 who underwent EMA/CO and EM/CO chemotherapy regimens. The model considered invasive mole, choriocarcinoma, clinical stage I, and presence of metastases as covariates. The analysis revealed that the use of EMA/CO and EM/CO (p=0.935), invasive mole (p=0.708), choriocarcinoma (p=0.996), and clinical stage I (p=0.622) were not significant predictors of chemoresistance. However, the presence of metastases emerged as an independent predictor of chemoresistance in women with a score ≤ 6 (OR: 6.54, 95% CI 1.57-27.20, p=0.010). This finding suggests that women with metastases are approximately 6.5 times more likely to develop chemoresistance compared to those without metastases, highlighting the importance of considering metastatic status when evaluating potential treatment resistance in this population.

**Figure 2:**
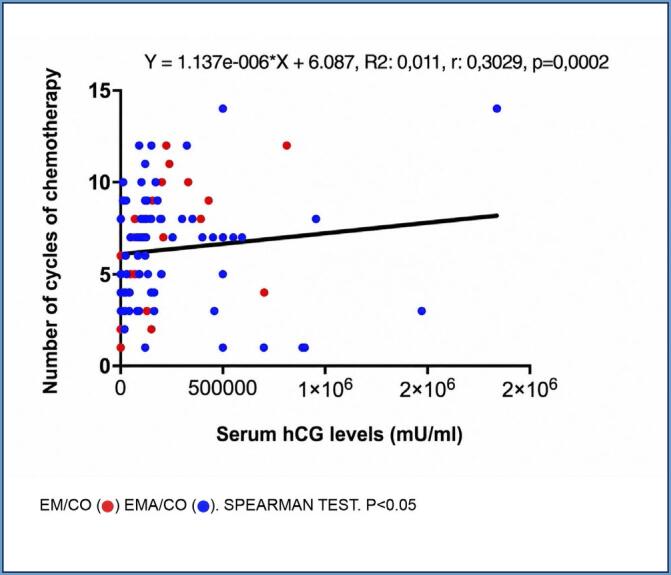
Correlation between the pre-treatment serum hCG levels and the number of chemotherapy cycles

A weak positive significant correlation (r=0.3029, p=0.0002) was observed between the pre-treatment serum hCG levels and the number of chemotherapy cycles. The model's coefficient of determination of 0.011 indicated that 1.1% of the variation in the number of chemotherapy cycles was linearly related to the pre-treatment serum hCG levels, while the remaining 98.9% of the variation was due to other factors not included in the model (Figure 2).

**Figure 3:**
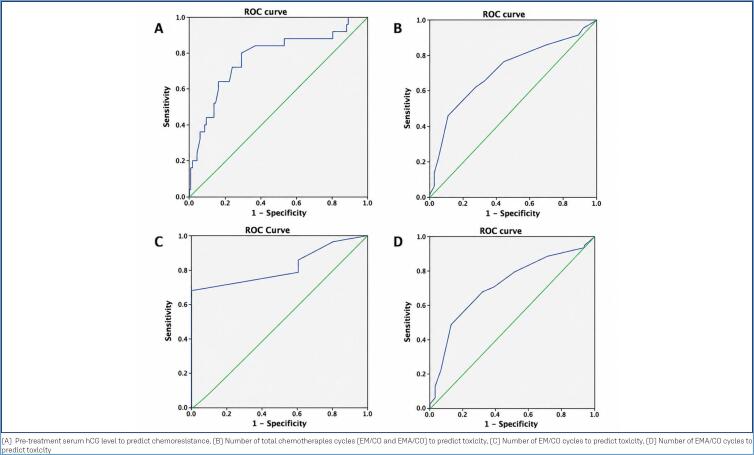
Receiver operating characteristics (ROC) curves

Considering all cases included in the study, a binary logistic regression model was created to assess whether pre-treatment serum hCG levels, pathology at diagnosis, history of pregnancy, type of chemotherapy regimen used during first-line treatment (EM/CO or EMA/CO), prognostic score (≤ 6 or ≥7) and presence of metastases were predictors of chemoresistance. It was observed that only pre-treatment serum hCG levels was a significant predictor of chemoresistance to chemotherapy [x2(1) =15.9, OR: 1.01, 95%CI: 1.00-1.01, R2 Nagelkerke 0.17, p=0.001]. The pre-treatment serum hCG levels was a moderately significant predictor (AUC: 0.77, 95%CI 0.66-0.88, p<0.0001) for identifying chemoresistance (Table 4S). A pre-treatment serum hCG levels of 149,133 mlU/ml was able to correctly identify 72.0% of women with GTN who developed chemoresistance to first-line treatment, with a false positive rate of 24.0% (Figure 3A) (Table 5).

**Table 5:** Pre-treatment serum hCG level cut-off value to predict chemoresistance and number of first-line cycles of treatment with EM/CO and EMA/CO to predict toxicity in women with gestational trophoblastic neoplasia

Variable	Cut-off value	AUC	95%CI	Sensitivity	Specificity	False positive	LHR +	LHR -	p-value
Pre-treatment serum hCG level (mlU/ml)	149,133	0.77	0.66-0.88	0.72	0.761	0.24	3.01	0.37	<0.0001
Number of total chemotherapies cycles (EM/CO and EMA/CO)	11.5	0.71	0.62-0.80	0.66	0.72	0.28	2.36	0.47	<0.0001
Number of EM/CO cycles	2.5	0.81	0.66-0.96	0.85	0.4	0.60	1.42	0.38	0.025
Number of EMA/CO cycles	6.5	0.71	0.61-0.81	0.68	0.68	0.32	2.13	0.47	<0.0001

AUC: area under ROC curve; CI: confidence interval; LHR+: positive likelihood ratio; LHR-: negative likelihood ratio

Considering all cases included in the study, a binary logistic regression model was created to assess whether the number of chemotherapy cycles during the first-line of treatment, not having received chemotherapy during the second-line of treatment, type of medication used during the second-line of treatment were predictors of toxicity. It was observed that only the number of cycles performed during the first-line of treatment [x2(1) =13.6, OR: 1.30, 95%CI: 1.12-1.52, R2 Nagelkerke 0.13, p=0.001] was a significant predictor of toxicity (Table 5S). The number of chemotherapy cycles was a moderately significant predictor (AUC: 0.71, 95%CI 0.62-0.80, p<0.0001) for identifying chemotherapy toxicity. The number of cycles value of 11.5 was able to correctly identify 66.0% of pregnant women with GTN who developed toxicity to the first-line of treatment, with a false positive rate of 28.0% (Figure 3B) (Table 5).

The number of EM/CO cycles was a strong significant predictor (AUC: 0.81, 95%CI 0.66-0.96, p<0.0001) for identifying toxicity to chemotherapy. The value of number of EM/CO cycles equal to 2.5 was able to correctly identify 85.0% of women with GTN who developed toxicity to EM/CO regimen with a false positive rate of 60.0% (Figure 3C) (Table 5).

The number of EMA/CO cycles was a moderately significant predictor (AUC: 0.71, 95%CI 0.61-0.81, p<0.0001) for identifying chemotherapy toxicity. The value of number of EMA/CO cycles equal to 6.5 was able to correctly identify 68.0% of women with GTN who developed toxicity to EMA/CO regimen with a false positive rate of 31.0% (Figure 3D) (Table 5).

## Discussion

In this study, we found that the modified EM/CO regimen was associated with higher rates of chemoresistance and need for second-line treatment compared to EMA/CO, particularly among high-risk GTN patients (score ≥ 7), while no significant differences were observed between regimens in low-risk patients.

Multiagent chemotherapy regimens are used to treat high-risk GTN. The most commonly used regimen is EMA/CO, and although the Cochrane Database Review was inconclusive as to which combination is superior, approximately 20% of women fail EMA/CO regimen, but most can be rescued with additional therapy.^(1)^ Overall survival rates for women with high-risk GTN now reach 95%.^(2)^ Although two other studies have already evaluated treatment options with actinomycin-D in low-risk GTN resistant to first-line treatment (3,4), there is no literature to date to support treatment with options such as actinomycin-D, or even its omission from the EMA/CO regimen, for low-risk GTN requiring multidrug therapy and for high-risk GTN requiring the EMA/CO regimen. Mora et al.^(18)^ compared remission rates between the three second-line regimens for low-risk methotrexate/folinic acid resistant GTN in a Brazilian cohort, observing similar remission rates between actinomycin-D (80%) and etoposide (71.4%), both of which were superior to carboplatin (47.8%).

In our study, we found a remission rate to EMA/CO of approximately 85.3%, with 14.7% of women showing chemoresistance, while in the group of women receiving EM/CO, the remission rate was 72.7%, with 27.3% of women showing chemoresistance, without statistical significance between the groups. As published in a previous study, remission rates to the EMA/CO regimen are between 71 and 78% and long-term survival between 85 and 94%.^(19)^ In high-risk GTN, the most commonly used regimen is EMA/CO, although a Cochrane Database Review was inconclusive as to which chemotherapy combination is superior.^(20)^ Approximately 20% of women fail EMA/CO regimen, but the majority can be rescued with additional therapy. Overall survival rates for women with high-risk GTN are close to 95%.^(21)^

Drug shortages are a global issue that can significantly impact cancer care. While the actinomycin-D shortage in Brazil has posed challenges in the management of GTN, similar issues have occurred internationally. For instance, the United States has faced shortages of carboplatin and cisplatin, critical drugs in treating various cancers. As highlighted by Gourd^(22)^, these shortages have delayed treatments and forced the use of less effective alternatives. This highlights the necessity for global collaboration to ensure the consistent availability of essential oncological drugs.

Women with a FIGO score ≥ 7 are at high-risk of developing drug resistance and are very unlikely to be cured with monotherapy. As a result, several different multidrug regimens have been developed.^(23,24)^ In the United Kingdom, the regimen consisting of EMA alternating with cyclophosphamide and vincristine (CO) every week has been developed. This regimen is widely used throughout the world because it appears to be effective with predictable and easily managed short-term toxic effects.^(25)^ The Korean Center for Gestational Trophoblastic Diseases found in a retrospective comparison that methotrexate/folinic acid had a remission rate of 63% (31 of 49 patients), methotrexate/folinic acid/dactinomycin 68% (27 of 40), methotrexate/dactinomycin/cyclophosphamide/doxorubicin/melphalan/hydroxycarbamide/vincristine 71% (32 of 45) and EMA/CO 91% (87 of 96).^(26)^ All of these regimens include actinomycin-D. Although two other studies have evaluated actinomycin-D treatment options for low-risk GTN resistant to first-line monotherapy^(12,18)^, there is no literature to date to support treatment with actinomycin-D options, or even omission of actinomycin-D from the EMA/CO regimen, for low-risk GTN requiring multidrug therapy and for high-risk GTN requiring the EMA/CO regimen.

When we evaluated women with a prognostic score ≥ 7, i.e. high-risk GTN, we found that women who used the EM/CO regimen without actinomycin-D had a higher prevalence of needing a second line and chemoresistance compared to women who used EMA/CO. The high recurrence rate observed in patients with a risk score ≥ 7 may be attributed to the intrinsic aggressiveness of high-risk GTN, greater tumor burden, presence of metastases, and the use of the modified EM/CO regimen lacking actinomycin-D, which may have contributed to suboptimal disease control in this subgroup. For women with a prognostic score ≤ 6, i.e. low- risk GTN, there was no significant effect of the chemotherapy regimen used in terms of number of cycles, need to use other lines of treatment, chemoresistance, toxicity, recurrence and death. These results reinforce the need for the complete regimen with actinomycin-D, EMA/CO, for high-risk GTN, in order to minimize chemoresistance rates and the need for a second line of treatment.

In our study, the pre-treatment serum hCG level was an independent predictor of chemoresistance in women with GTN. Singhal et al.^(27)^ performed a retrospective study with 116 women, being 60 low- and 56 high-risk GTN. Risk of chemotherapy resistance was higher in women with intermediate-risk score (5-6), and risk of recurrence was more in those with ultra-high risk score (≥ 13). Age, myometrial invasion, serum beta-hCG and tumor size were not related to chemoresistance or recurrence. In agreement with our results, Weng et al.^(28)^ retrospectively analyzed data from 578 GTN women who received chemotherapy. The authors observed that pretreatment serum hCG level and interval from previous pregnancy were independent predictors of both first-line and subsequent single-agent chemoresistance. In an international multicenter study involving 431 women with GTN presenting with a FIGO risk score of 5 or 6 with a minimum of 2 years of follow-up, univariable and multivariable logistic regression revealed that metastatic disease, choriocarcinoma histology, and pretreatment hCG level were significant predictors of resistance to single-agent therapies.^(29)^

We observed that only the number of cycles given during the first line of treatment was a significant predictor of toxicity in both the EMA/CO and the modified EM/CO group. Late toxicity could not be assessed in this study because women were followed for up to 1 year after completion of treatment with EMA/CO and EM/CO. However, the potential risk of chemotherapy-induced second cancers is very low. The largest GTN study to date, with more than 30,000 patient-years of follow-up, reported no overall increase in the risk of second cancers in women treated with methotrexate or EMA/CO alone.^(30)^ Wu et al.^(31)^ analyzed retrospective women with low-risk GTN administered actinomycin-D salvage therapy after failing methotrexate chemotherapy. The final analysis included 89 cases. Of these, 73 cases (82.02%) responded to salvage actinomycin-D. The remaining 16 resistant cases were switched to etoposide/methotrexate/actinomycin-D/cyclophosphamide/vincristine chemotherapy and achieved complete remission. Except for 2 cases requiring other salvage regimens due to actinomycin-D toxicity, 97.80% of cases tolerated the toxicity.

## Conclusion

In this study, while outcomes were similar between regimens in low-risk GTN, the use of the modified EM/CO regimen in high-risk patients was associated with higher rates of chemoresistance, recurrence, and need for second-line treatment compared to the standard EMA/CO regimen. Pre-treatment serum hCG levels and number of chemotherapy cycles were moderately significant predictors for chemoresistance and toxicity, respectively.

## Data Availability

The research data are described in the article presented.
