# Supplementary material for: Impact of different EMA/CO treatment regimens in women with gestational trophoblastic neoplasia: brazilian multicenter retrospective cohort study
Source: Rev Bras Ginecol Obstet. 2026 Jul 17;48:e-rbgo52. doi: 10.61622/rbgo/2026rbgo52 (PMC13399383; doi:10.61622/rbgo/2026rbgo52)
Supplement: Supplementary Material [file 1806-9339-rbgo-48-e-rbgo52-Suppl01.pdf]

Supplementary material

Table 1S. Chemotherapy regimens with EMA/CO and EM/CO

Day	Drugs	Dose
EMA/CO		
1	Etoposide<br>ActDMTX MTX	100 mg/m² diluted in 200 ml of saline solution and infused IV over 30 min<br>0.5 mg IV bolus<br>100 mg/m² IV bolus<br>200 mg/m² by IV infusion over 12h
2	Etoposide<br>ActD<br>Folinic acid	100 mg/m² diluted in 200 ml of saline solution and infused IV<br>over 30 min<br>0.5 mg IV bolus<br>15 mg IM every 12h (4 doses) or oral route starting 24h after starting MTX
8	Cyclophosphamide<br>Vincristine	600 mg/m² diluted in saline solution and infused over 30 min<br>1 mg/m² bolus IV
EM/CO		
1	Etoposide<br>Cisplatin	150 mg/m² diluted in 200 ml of saline solution and infused IV<br>over 30 min<br>75mg/m² IV with prior hydration
8	Etoposide<br>MTX<br>ActD<br>Folinic acid	100mg/m² diluted in 200 ml of saline solution and infused IV over 30 min<br>300mg/m² by IV infusion over 12h<br>0.5 mg IV bolus<br>15 mg IM every 12h (4 doses) or oral route starting 24 h after starting MTX

ActD: actinomycin-D, MTX: methotrexate

Table 2S. Association between chemotherapy regimen used and toxicity and main side effects

	EM/CO (N=33)	EMA/CO (N=109)	p-value
Toxicity	84.8% (28/33)	71.6% (78/109)	0.124 *
Anemia	63.6% (21/33)	69.7% (76/109)	0.510 *
Neutropenia	33.3% (11/33)	24.8% (27/109)	0.330 *
Neuropathy	21.2% (7/33)	12.8% (14/109)	0.235 *
Nausea and vomiting	60.6% (20/33)	56.9% (62/109)	0.704 *
Thrombocytopenia	9.1% (3/33)	10.1% (11/109)	0.866 *
Stomatitis	45.5% (15/33)	46.8% (51/109)	0.893 *
Creatinine elevation	3.0% (1/33)	7.3% (8/109)	0.373 *
Altered liver function	21.2% (7/33)	22.9% (25/109)	0.836 *
Diarrhea	18.2% (6/33)	11.0% (12/109)	0.278 *
Use of granuloquine	54.5% (18/33)	70.6% (77/109)	0.085 *

Chi-squared *: % (n/N). p<0.05

Table 3S. Influence of the chemotherapy regimen used during the first-line treatment in women with a score ≤ 6

	EM/CO (N=20)	EMA/CO (N=49)	OR (95%CI)	p-value
Number of cycles (first-line treatment)	4.8 (2.6)	6.0 (2.4)		0.065 *
Second-line treatment	10.0% (2/20)	16.3% (8/49)	0.56 (0.11-2.88)	0.712 **
Chemoresistance	10.0% (2/20)	16.3% (8/49)	0.56 (0.11-2.88)	0.712 **
Recurrence	10.0% (2/20)	8.2% (4/49)	1.25 (0.22-5.75)	> 0.999 **
Toxicity	80.0% (16/20)	69.4% (34/49)	1.76 (0.51-5.46)	0.553 **
Death	0.0% (0/20)	2.0% (1/49)	0.0 (0.0-22.05)	> 0.999 **

OR: odds ratio; CI: confidence interval. Student's t-test *: mean (standard deviation); Chi-squared **: % (n/N); binary logistic regression. p<0.05

Table 4S. Odds ratio for chemoresistance to first-line chemotherapy treatment

Variable	OR	95%CI	p-value
Pre-treatment serum hCG levels	1.01	1.00-1.01	0.001
Pathology in diagnosis			
Invasive mole	0.39	0.11-1.41	0.154
Choriocarcinoma	2.29	0.90-5.85	0.810
Previous pregnancy			
Molar pregnancy	1.29	0.47-3.52	0.610
Miscarriage	0.00	0.00-Infinite	0.998
Ectopic pregnancy	0.27	0.29-79.99	0.271
Full-term pregnancy	1.47	0.48-4.44	0.495
Use of EM/CO	2.18	0.86-5.53	0.101
Use of EMA/CO	0.46	0.18-1.16	0.101
Prognosis score ≤ 6	0.65	0.27-1.58	0.346
Prognosis score ≥ 7	1.52	0.63-3.67	0.346
Metastases	2.44	0.94-6.28	0.064

OR: odds ratio; CI: confidence interval. Binary logistic regression. p<0.05

Table 5S. Odds ratio for toxicity to chemotherapy treatment

Variable	OR	95%CI	p-value
Number of cycles (first-line treatment)	1.30	1.12-1.52	0.001
Second-line treatment			
No chemotherapy	0.00	0.00-infinite	0.998
EP/EM	infinite	0.00-infinite	0.999
EP/EM + TE/TP	infinite	0.00-infinite	0.999
EP/EMA	infinite	0.00-infinite	0.999
EP/EMA + TE/TP	infinite	0.00-infinite	0.999
TE/TP + ICE	infinite	0.00-infinite	0.999
TE/TP + Pembrolizumab	infinite	0.00-infinite	0.999

OR: odds ratio; CI: confidence interval. Binary logistic regression. P<0.05. EP/EM: etoposide, platinum, etoposide and methotrexate; TE/TP: paclitaxel, etoposide, paclitaxel and platinum; EP/EMA: etoposide, platinum, etoposide, methotrexate and actinomycin-d; ICE: ifosfamide, cyclophosphamide and etoposide
